# Primary care physicians’ perceptions of Israel’s national program for quality indicators in community healthcare– 2010 and 2020

**DOI:** 10.1186/s13584-025-00685-5

**Published:** 2025-05-02

**Authors:** Rachel Nissanholtz-Gannot, Ayala Burger, Bruce Rosen

**Affiliations:** 1 Department of Health Systems Management, School of Health Sciences, Ariel University, Ariel, Israel; 2 Smokler Center for Health Policy Research, Myers-JDC-Brookdale Institute, Jerusalem, Israel

**Keywords:** Quality indicators, Measurement, Primary care physicians, Community health services

## Abstract

**Background:**

Monitoring the quality of primary care is essential for improving healthcare services. The National Program for Quality Indicators in Community Healthcare measures various aspects of healthcare quality. A 2010 survey among Israeli primary care physicians (PCPs) found widespread support for the program alongside concerns about its effects on workload and competitiveness. This study assessed the extent to which PCPs’ perceptions had changed between 2010 and 2020.

**Methods:**

Cross-sectional survey on PCP’s experience with the quality monitoring effort at their health maintenance organizations were conducted in 2010 and 2020 among representative samples of PCPs. Bivariate analysis examined whether the study variables varied between the timepoints. Logistic regression models evaluated the extent to which the participants’ characteristics and perceptions contribute to their attitudes toward the program.

**Results:**

The study sample comprised 605 physicians in 2010 and 450 physicians in 2020. Overall, support for the National Program for Quality Indicators was high in both surveys. However, between 2010 and 2020 some decrease in the support for the use of quality indicators was observed among PCPs The greatest decrease in support between 2010 and 2020 was observed in the proportion of respondents who perceived that it is important to a great or very great extent to measure the clinical performance of some quality indicators (88% versus 81%) and in the proportion of respondents who perceived that monitoring contributed to improvement (66% versus 60%). Over half of respondents (58%) perceived to a large or very large extent that the program was associated with increased workload compared to 63% in 2010. Similar proportions of respondents in 2010 and 2020 felt that the program was also associated to a large or very large extent with over-competition (47% and 48%, respectively) and excess managerial pressure (58% and 60%, respectively).

**Conclusions:**

The study indicates that while support for the program in general remains high, it continues to have undesirable side effects. Further use of the program for quality indicators must consider the shortcomings voiced in 2010 which have remained uncorrected as reflected in the results of the 2020 survey: extreme managerial pressures, increased workload and over-competitiveness.

## Background

Healthcare quality indicators are used in many countries to assess, and ultimately improve the quality of healthcare services [1–5]. The National Program for Quality Indicators in Community Healthcare, which was established in Israel in 2000, collects data on 73 indicators provided in the community, most of which are process indicators (https://www.israelhealthindicators.org/). The program relies on the voluntary participation of Israel’s four public health maintenance organizations (HMOs) [6–8], and mainly uses large-scale computerized databases maintained by these HMOs, to assess the quality of selected services [9]. The program currently covers nine areas of measurement (health promotion, cancer screening, child and adolescent health, adults over 65, respiratory diseases, cardiovascular health, diabetes, antibiotic treatment, and mental health). The data enables ongoing, continuous, and dynamic monitoring and provides information to policymakers and the public [2, 5–7]. Among the four HMOs, at least the two largest ones, covering about 80% of the population, have added additional internal indicators. However, to the best of our knowledge, physicians are not aware which indicators are National Program ones, and which are internal.

Since the inception of the program, studies have indicated improvement in several quality indicators. For example, increased immunization rates among the elderly and improved healthcare of the elderly population [10], a growth in the use of community-based healthcare services [11], and a significant increase in screening for breast cancer and colorectal cancer [12]. Furthermore, longitudinal adherence to quality indicators in diabetes care was found to be associated with reduced risk of cardiac morbidity [13].

The opinion of healthcare providers, and their stance regarding quality monitoring programs have a major effect on the success of such programs [6]. Healthcare providers in the United Kingdom have consistently expressed a positive view of quality monitoring programs, both in hospitals and in the community [14, 15]. A similar approach has been voiced by healthcare providers in Israel [16, 17]. In 2010, some of the researchers involved in the present study, conducted a survey among PCPs working in the four HMOs regarding their experience with the quality monitoring effort. The results of the survey showed that most respondents (87%) felt that quality monitoring by indicators was important and many of them (72%) supported the program’s continuation [17]. On the other hand, 60% noted they felt extreme managerial pressures due to the program, and 65% mentioned that they must cope with increased workloads. 40% of the respondents criticized the over-competitiveness generated by the program [17]. In the years following the 2010 study, changes were made to the National Program for Quality Indicators in Community Healthcare. These included the publication of comparisons of indicators among the four HMOs. To alleviate the burden on physicians, the number of national indicators that measure physician performance, which may increase the competition among HMOs, were reduced. HMOs continued to collect internal metrics on physician performance [personal discussion with HMO managers]. These changes called for reconsidering the implications of the program and reevaluating the viewpoints of healthcare providers on the issue, including their extent of support of the program. To that end, we conducted a second survey in 2020 to examine the views of PCPs on the program.

## Methods

### Setting and participants

Cross-sectional surveys were conducted in 2010 and 2020 among representative samples of PCPs working in the four public HMOs. The results of the 2010 survey were previously published [17].

The study population consisted of PCPs working for the HMOs (full- or part-time, salaried or self-employed contractors) engaged in the direct care of adult patients. Physicians with no responsibility for the quality of care for a panel of patients (i.e., consultants, physicians engaged mainly in administrative or managerial work, retired physicians, and temporary replacements) were excluded from the study population. The study team estimated that approximately 4400 Israeli physicians met these criteria. For each survey, each HMO provided the contact details of a sample of PCPs randomly selected from their administrative records. After receiving the lists from the HMOs comprising 1000 PCPs in 2010 and 896 PCPs in 2020, the eligibility criteria were checked again and PCPs who did not fulfill them were excluded. This resulted in 804 PCPs who met the criteria and were approached in 2010 and 725 PCPs who met the criteria and were approached in 2020.

The study was approved by Myers-JDC-Brookdale’s institutional ethics committee (approval number IRB-BH-261). All participants provided their consent to participate in the study and were assured anonymity.

### Study questionnaire

The development of the study questionnaire for the 2010 survey was previously described [17]. The development process included an internal validation, a pilot study and revision following comments received in the pilot study. The same questionnaire was used in the 2020 survey. In total, the study questionnaire comprised 105 questions, of which 22 were open-ended.

The questionnaire addressed physicians’ experience with the quality monitoring effort at their HMOs. The main topics of the questionnaires included PCPs’ experiences with the program; their perceptions about the quality indicators and definitions; their assessment of its impact on their work, patient care, and their relationship with their patients, their colleagues, and their health plans; their difficulties and concerns regarding the program; suggestions for improving the program; use of the information gathered through the program; satisfaction with the program and desires regarding its future. Information on respondents’ personal and professional characteristics was also collected.

Responses to each study item were provided on a six-point scale ranging from 1 (very little or not at all) to 6 (to a very large extent).

### Data collection

The 2010 survey took place between August and December 2010. The 2020 survey took place between December 2019 and February 2020. Sampled PCPs were approached by email (2020) or regular post (2010) and had an opportunity to respond either by telephone, regular post, or fax (in 2010) or by email or telephone (in 2020). Designated respondents received up to four reminders by telephone or email. In 2010, most respondents (85.7%) replied by phone, regular post or fax, whereas 14.3% of respondents replied by email. In 2020, most participants (81.5%) replied by email, and the rest (18.5%) replied by other means (*p* < 0.001 for the difference between the survey years).

### Response rate

Only questionnaires with complete information on demographic and professional characteristics were included in the data analysis. Of 804 PCPs who met the eligibility criteria and were approached for participation in 2010, 605 (75.2%) provided complete questionnaires. Of 725 PCPs approached in 2020, 450 (62.1%) provided complete questionnaires, including information on demographic and professional characteristics.

### Data analysis

Analyses were conducted using the Statistical Package for the Social Sciences (SPSS), version 24 (IBM, Armonk, NY, USA).

Non-responses to the closed-ended questions were treated as missing values. Variables were compared by chi-square test.

Bivariate analysis was performed to examine whether the study variables varied across key subgroups of PCPs and between and between the two survey time points. Logistic regression models were performed to assess the extent to which the participants’ characteristics and perceptions contribute to their attitudes toward the monitoring program. The results were presented as odds ratio with 95% confidence interval (CI) [18].

The data were weighted to reflect the differences among the HMOs in their size (i.e., the number of PCPs working at each HMO) and response rates (HMO-specific response rates ranged from 59 to 67%) so that the results would more accurately reflect the national study population. The weighting also considered the relationship between the sampling probability and the number of HMOs in which each PCP worked (i.e., a PCP working for two HMOs was more likely to be included in the sample than a PCP working for only HMO). All statistical analyses were conducted by using the weights.

As not all respondents provided all answers to all questions, some regression models included less than 1055 responses. There was no imputation of missing values.

P-value < 0.05 was considered statistically significant.

## Results

### Demographic and professional characteristics of the study sample

Comparison of respondent characteristics by survey year showed differences in age categories, country of birth, medical specialty, type of employment and main type of practice (Table 1). A larger percentage of the study population in 2020 was over 45 years of age compared to 2010, and a larger percentage was non-Jewish. Additionally, in 2020, a greater proportion of internal doctors/other specialists and a smaller proportion of family physicians and non-board-certified physicians comprised the study population compared to 2010. At each timepoints, a similar proportion of respondents were specialists in family medicine and physicians without board certification. Specialists in internal medicine and other fields who work as PCPs comprised about a fifth of the study population. In 2010 about a quarter of the respondents worked as independent physicians, in 2020 this type of employment was reported by over a third of the respondents.

**Table 1:** Demographic and professional characteristics of the study population by survey year (%)

Characteristic	2010 *N* = 605 *n* (%)	2020 *N* = 450 *n* (%)	*P* value*
**Age category**,** years**			< 0.001
< 44	148 (24.5)	75 (16.6)	
45–60	343 (56.7)	224 (49.8)	
> 60	114 (18.8)	151 (33.6)	
**Sex**			NS
Female	263 (43.5)	182 (40.4)	
Male	342 (56.5)	268 (59.6)	
**Ethnicity**			NS
Jewish	462 (76.4)	319 (71.0)	
Non-Jewish	143(23.6)	131 (29.0)	
**Country of birth**			< 0.001
Israel	236 (39.0)	236 (52.4)	
Other	369 (61.0)	214 (47.6)	
**Specialty**			< 0.001
Family medicine	246 (40.6)	160 (35.5)	
Internal medicine\other	122 (20.2)	133 (29.6)	
Not board certified	237 (39.2)	157 (34.9)	
**Type of employment**			< 0.001
Salaried only	287 (47.4)	189 (41.9)	
Independent only	167 (27.7)	172 (38.3)	
Both	151 (24.9)	89 (19.8)	
**Main type of practice**			< 0.001
Primary care	553 (91.4)	368 (81.8)	
Specialist	52 (8.6)	82 (18.2)	

*p by chi-squared test for the difference between 2010 and 2020

NS = not statistically significant

### PCPs’ perceptions on the National program and the quality indicators collected

**Fig. 1:**
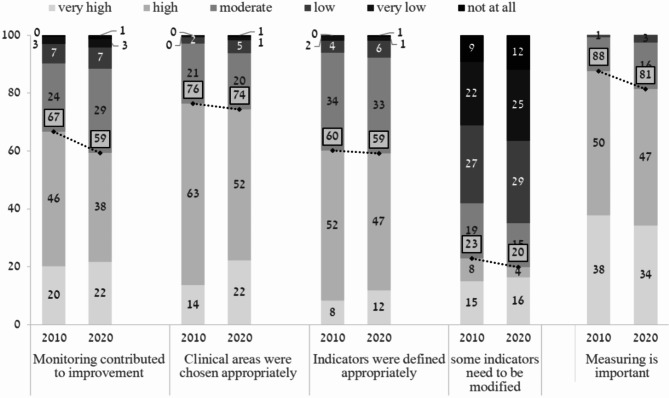
PCP perceptions on the National Program for Quality Indicators in Community Healthcare and the quality indicators collected: comparisoדn between responses in the 2010 and 2020 surveys. Responses were provided on a scale of 1 (to a very small extent) to 6 (to a very large extent). * The difference between the years is statistically significant. The numbers in white squares indicate the percentage of responders who responded “to a very high” and “high” extent

Overall, support for the National Program for Quality Indicators was high at both time points surveyed. However, in 2020, these proportions statistically significantly decreased compared to 2010. Most physicians perceived that monitoring clinical performance is important to very important, with a statistically significant lower proportion of physicians agreeing with this statement in 2020 compared to 2010 (88% versus 81%, *p* = 0.03). Two-thirds of physicians (66%) surveyed in 2010 compared to 60% of physicians surveyed in 2020 thought that monitoring contributes to improved quality to a great or very great extent, but this difference was not statistically significant (NS). A higher proportion of physicians surveyed in 2010 compared to 2020 expressed their support for continuing the program (73% versus 65%, *p* = 0.03). A higher proportion of physicians surveyed in 2010 compared 2020 perceived that the program increases workload to a great or very great extent (63% versus 58%, NS). Less than half of physicians replied that the program affected their satisfaction with their job to a great or very great extent, with a statistically significantly higher proportion of physicians expressing increased satisfaction in 2010 compared to 2020 (48% versus 37%, *p* = 4.5*10^− 5^) (Fig. 1; Table 2). The proportions of physicians who believed to a great or very great extent that the clinical areas were chosen appropriately were similar at both time points (76% and 74% in 2010 and 2020, respectively, NS) as was the proportion of physicians who believed that the indicators were defined appropriately (60% and 59% in 2010 and 2020, respectively, NS).

**Table 2:** The relationship between the main study variables and physician demographics and professional characteristics by survey year

	Monitoring clinical performance is important^1^			Monitoring contributes to improved quality^2^		Support continuing the program^3^		The program increases workload^4^		Job satisfaction related to the program^5^	
	2010 M = 605 %		2020 *N* = 450 %	2010 M = 605 %	2020 *N* = 450 %	2010 M = 605 %	2020 *N* = 450 %	2010 M = 605 %	2020 *N* = 450 %	2010 M = 605 %	2020 *N* = 450 %
**Total**	88*		81*	67	59	73*	65*	63	58	48***	37***
	(0.03)					(0.03)				(4.5*10^− 5^)	
**Age**											
< 44	87		91	58	59	69	64	62	62	36	32
45–60	89***		75**	70**	56**	73***	57***	64	59	50**	36**
	(1.5*10^− 4^)			(0.009)		(0.0099)				(0.002)	
> 60	85		86	68	64	77	76	62	54	52	40
**Sex**											
Female	87		80	67	59	72**	58**	59	57	42	32
						(0.008)					
Male	88		82	64	60	71	70	67	59	50***	39***
**Population group**										(1.1*10^− 4^)	
Jewish	96		94	78	77	83	81	67	58	69**	58**
										(0.004)	
Non-Jewish	85**		76**	63	52	69**	58**	62	58	40**	27**
	(0.003)					(0.01)				(0.005)	
**Country of birth**											
Israel	88**		78**	67	55	74	68	59	56	47	36
	(0.004)										
Other	87		84	66	64	69	63	71***	60***	48***	37***
**Specialty**								0.001		(4*10^− 4^)	
Family medicine	80*		71*	58	47	62**	45**	73	66	35***	15***
	(0.03)					(0.003)				(1*10^− 4^)	
Internal medicine\Other	93		80	68	55	73	61	55	52	47	37
Not board certified	93		93	75	76	83	89	57	55	60	57
**Type of employment**											
Salaried only	83		80	66	60	71	68	59	64	47*	39*
										(0.04)	
Independent only	95***		78***	70	56	76*	63*	57***	47***	46*	34*
	(5.9*10^− 4^)					(0.04)		(2.8*10^− 4^)		(0.04)	
Both	88		89	64*	62*	71	63	78	67	48	35
				(0.03)							
**Main type of practice**											
Primary care	87		82	66*	60*	72	67	65	59	48***	38***
				(0.03)						(8.3*10^− 5^)	
Specialist	92*		75*	68	56	74	52	50	53	38*	34*
	(0.02)									(0.02)	

* p value = 0.05 − 0.01

** p value = 0.01 − 0.001

*** p value < 0.001

^1^important / very important; ^2^to a great or very great extent; ^3^yes; ^4^to a great or very great extent; ^5^satisfied / very satisfied

At the same time, one fifth of the respondents (20%) in 2020 recommended modifying some of the specific indicators to a great or very great extent, and this was lower than the proportion in 2010 (23%) (Fig. 1). 7% of respondents mentioned that there are some unnecessary clinical areas included in the program, like asthma, vaccinations, and diabetes, alongside some missing clinical areas such as health promotion, cancer and early detection of cancer, osteoporosis, and the quality of communication with patients. The most mentioned unnecessary and missing clinical areas in the 2020 survey were similar to those mentioned in 2010.

Comparison of the responses provided by family medicine specialists, internal medicine/other specialists and physicians who are not board certified (Table 2), showed that between 2010 and 2020, there was a statistically significant decrease in the proportion of family medicine specialists who perceived that monitoring clinical performance is important, supported the continuation of the program, and reported that the program increased their job satisfaction to a great/very great extent (*p* < 0.05, *p* < 0.01 and *p* < 0.001, respectively). No statistically significant change was observed in these parameters for the other physician subgroups. Notably, in 2020, the proportion of family physicians who reported that the program increased their job satisfaction to a great/very great extent was very low (15%) compared to the other physicians.

### Perceived challenges associated with the program

**Fig. 2:**
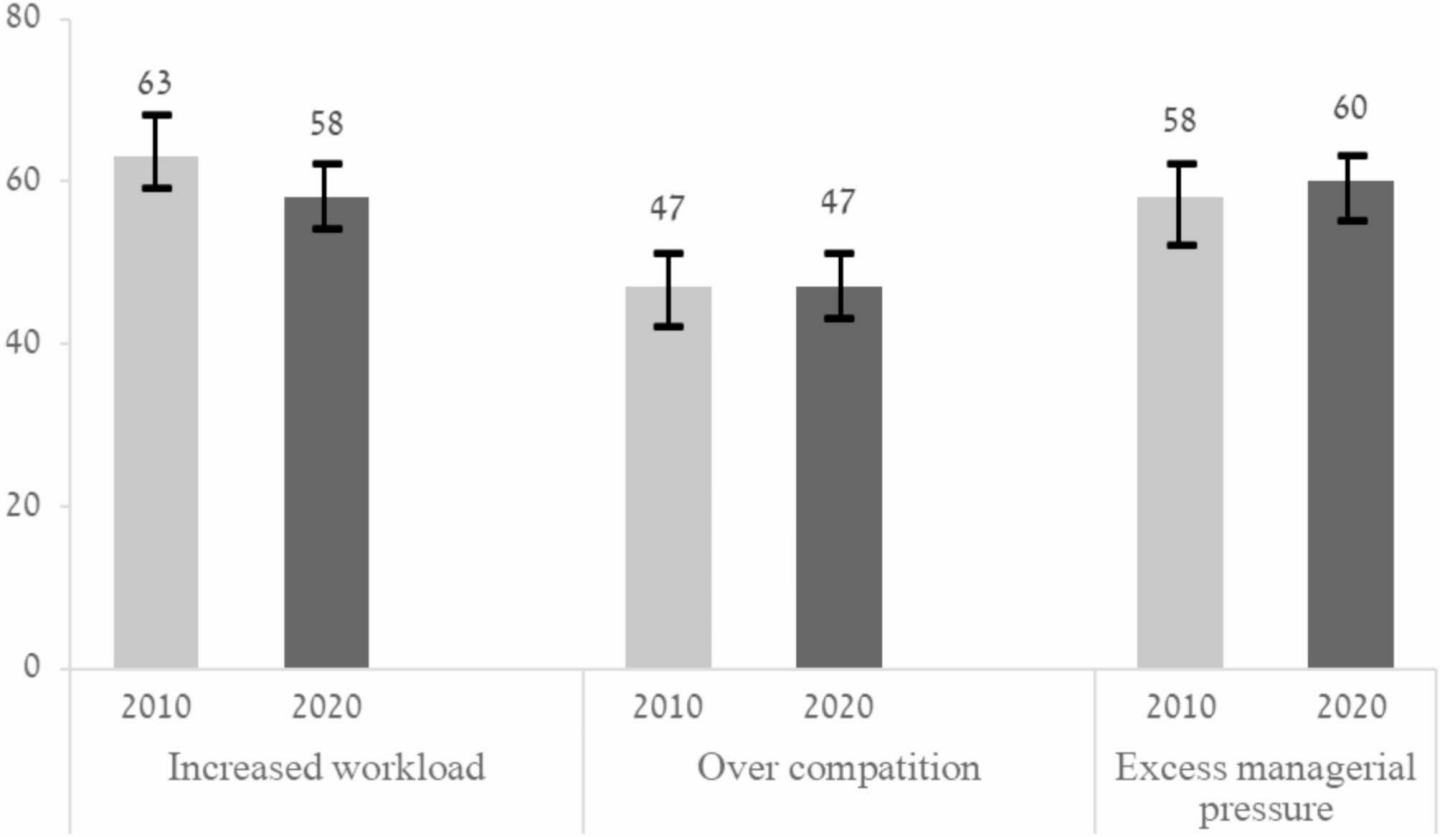
Challenges related to the National Program for Quality Indicators in Community Healthcare. The bars depict the percentage of PCPs who rated the challenge as “high” or “very high”, by survey year (%). *Note: The differences are not statistically significant

Over half of respondents perceived that the program was associated with increased workload (63% in 2010 and 58% in 2020), over-competition (47% and 48%, respectively) and excess managerial pressure (58% and 60%, respectively), but the differences between the years were not statistically significantly different (Fig. 2).

The most common changes suggested by the respondents for handling these challenges included emphasizing outcome rather than process indicators; reducing the number of indicators; providing advanced training, using automatic measuring tools; ending the practice of sharing comparisons between HMOs with the public; ending the practice of sharing comparisons among physicians within HMOs; reducing organizational pressures; and allocating specific time slots for quality measurement.

In both surveys more than half of the respondents perceived to a great/very great extent that HMOs did their best to help them improve their performance in quality indicators (51% in 2010 and 54% in 2020, NS). When asked to identify the main changes they would make in the way that HMOs should learn from the indicators, the most common responses were providing more job positions for healthcare personnel (physicians, nurses, secretaries, health promoters etc.), expanding the visiting time allocated for each patient, increasing the availability of laboratory and other tests (such as magnetic resonance imaging, computed tomography, mammography, etc.) and the availability of specialists. In addition, they suggested expanding the dedicated time allocated for dealing with quality indicators and raising physicians’ salary.

### Physicians’ job satisfaction

Many of the physicians who participated in the survey in 2020 indicated that they were generally satisfied or very satisfied with their work (84%, compared to 80% in 2010), 13% were moderately satisfied, and 3% were unsatisfied or very unsatisfied. In 2020, more than a third of respondents (37%) compared to 48% in 2010 reported increased job satisfaction since the monitoring program was implemented (*p* = 4.5*10^− 5^). Furthermore, two-thirds of respondents (66% in 2010 and 63% in 2020) felt satisfied or very satisfied with their performance measured by the quality indicators, as compared to the other physicians in their districts. The difference was not statistically significant between the survey years.

### Correlates of physicians’ perceptions

Bivariate analysis was performed to examine whether the study variables varied across key subgroups of respondents and across study years. As shown in Table 2, PCPs over 60 years of age, male, Jewish, not board-certified supported the continuation of the program more than younger PCPs, female, non-Jewish, board certified and specialists. Nonetheless, in almost all *of* these subgroups, support decreased for the program in its current setup between 2010 and 2020.

In almost all strata, respondents to the 2020 survey reported less burden at work compared to the 2010 respondents. This decrease is shown clearly among PCPs who were born abroad, and among independent physicians and those who hold salaried together with independent position. In contrast, the sense of workload increased among PCPs who work as salaried physicians only and among specialists.

Despite a slight decrease between 2010 and 2020, most respondents in all strata supported the continuation of the indicators program.

### Covariates of PCP perceptions

Table 3 presents the results of logistic regressions, which assessed the independent effects of a variety of personal and professional characteristics on PCP attitudes toward the monitoring program. This subgroup analysis showed that non-Jewish PCPs versus Jewish PCPs, females versus males, and PCPs without a board certification compared to board certified PCPs were more likely to perceive that the program improves quality and that it is important. Other demographic and personal characteristics had a mixed effect on PCPs’ attitudes towards the program.

**Table 3:** Logistic regressions of selected outcome variable related to the National program for quality indicators in community healthcare on PCPs’ personal and professional characteristics*

	Odds ratio (95% confidence interval)					
	Monitoring performance is important ^1^	Monitoring contributes to improved quality ^2^		Support continuing the program ^3^	The program increases workload ^4^	Job satisfaction related to the program ^5^
**Age** (Reference group: Age < 45)						
45–60	**0.47 (0.28–0.81)** **(p value = 0.006 )**	1.32 (0.92–1.90)	0.71 (0.44–1.14)		1.17 (0.81–1.68)	**1.64 (1.11–2.42)** **(p value = 0.01 )**
> 60	0.71 (0.38–1.33)	**1.64 (1.07–2.51)** **(p value = 0.02 )**	1.23 (0.70–2.17)		0.98 (0.64–1.49)	**1.82 (1.16–2.87)** **(p value = 0.009 )**
**Born in Israel**	0.92 (0.62–1.39)	1.17 (0.85–1.61)	0.86 (0.57–1.30)		1.15 (0.83–1.60)	0.71 (0.49–1.02)
**Jewish**	**0.26 (0.15–0.47)** (p value = 7.5*10 ^**− 6**^ **)**	**0.3 (0.24–0.52)** (p value = 1.3*10 ^**− 7**^ **)**	**0.41 (0.25–0.67)** (p value = 3.8*10 ^**− 4**^ **)**		1.24 (0.86–1.79)	**0.20 (0.13–0.29)** (p value = 1.8*10 ^**− 15**^ **)**
**Male**	**0.67 (0.45–0.99)** **(p value = 0.05 )**	**0.65 (0.48–0.88)** **(p value = 0.006 )**	0.86 (0.58–1.27)		1.29 (0.95–1.75)	0.97 (0.70–1.34)
**Board certification** (Reference group: Not board certified)						
Family physician	**0.36 (0.22–0.59)** (p value = 5.1*10 ^**− 5**^ **)**	**0.46 (0.32–0.65)** (p value = 1.2*10 ^**− 5**^ **)**	**0.36 (0.23–0.58)** (p value = 1.9*10 ^**− 5**^ **)**		1.24 (0.88–1.76)	**0.37 (0.25–0.52)** (p value = 4.1*10 ^**− 8**^ **)**
Internist and other	0.71 (0.41–1.24)	**0.59 (0.40–0.85)** **(p value = 0.005 )**	**0.44 (0.26–0.72)** **(p value = 0.001 )**		0.88 (0.62–1.27)	0.86 (0.59–1.24)
**Work primarily as specialist**	**1.74 (1.02–2.97)** **(p value = 0.04 )**	0.98 (0.64–1.49)	1.25 (0.73–2.12)		1.33 (0.88–2.03)	1.57 (0.99–2.48)
**Mode of employment** (Reference group: Independent only)						
Salaried only	0.71 (0.42–1.20)	1.05 (0.72–1.52)	1.18 (0.71–1.97)		1.11 (0.77–1.59)	1.35 (0.92–1.97)
Salaried and Independent	1.68 (0.90–3.12)	1.03 (0.68–1.58)	1.01 (0.59–1.75)		**1.60 (1.05–2.44)** **(p value = 0.03 )**	1.16 (0.75–1.81)
**Response via Email**	1.22 (0.74-2.00)	1.05 (0.73–1.51)	0.80 (0.50–1.28)		0.81 (0.56–1.17)	0.80 (0.55–1.18)
**Study year 2020**	**0.52 (0.32–0.87)** **(p value = 0.01 )**	**0.65 (0.45–0.94)** **(p value = 0.02 )**	0.85 (0.53–1.36)		0.93 (0.64–1.34)	**0.65 (0.44–0.95)** **(p value = 0.03 )**
Cox & Snell R^2^	**0.12**	**0.09**	**0.17**		**0.09**	**0.19**
Nagelkereke R^2^	**0.21**	**0.13**	**0.24**		**0.12**	**0.26**
N (unweighted)	1022	1017	909		1017	1015

^1^ Important or very important; ^2^ To a great or very great extent; ^3^ Yes; ^4^ To a great or very great extent; ^5^ Satisfied or very satisfied

* The model also included data about the HMO in which the PCP works

Bold indicates significance at *p* < 0.05

## Discussion

Two main findings emerge from the 2020 survey conducted among PCPs to examine their perceptions of the Israel National Program for Quality Indicators in Community Healthcare. First, most respondents think that the program is important, contributes to the quality of medical care and they support its continuation. However, although a high percentage of PCPs supported the program in 2020, the support level was slightly lower than it was in 2010. Second, it seems that the steps taken to mitigate the side effects of the program (e.g., reducing workload, excessive managerial pressure and over competition) had little effect or were offset by other developments. Despite changes made in the program, PCPs’ perceptions of the program’s adverse effects did not change substantially, except for a small but significant decrease in the proportion of PCPs who felt that the quality monitoring program increased their workload to a great extent.

It is possible that the actions of HMO managements were not felt by PCPs. In addition, several changes occurred in the decade between the two surveys that may have offset internal efforts to make the measurement program less onerous. One such change was the publication of inter-HMO comparative data starting in 2012. That change alone may have increased the workload and pressure perceived by PCPs. However, our study has shown that the proportion of PCPs reporting stress and competition did not increase between 2010 and 2020. This observation may be attributed to the HMOs’ actions to reduce the program’s significant challenges which may have offset PCPs’ perceived pressure due to the publicizing of indicators.

In contrast, to physicians without board certification, who perceived greater benefit and support to the quality indicators program, family medicine specialists showed the greatest objections to the program. This group of professionals have trained and passed boards in this specific field of primary care medicine and are the only ones who train students and trainees in the clinics. Therefore, they may perceive the quality indicators program as a threat to their autonomy. We observed similar trends of reduced support for the program, reduced work satisfaction and decreased perception that monitoring contributes to improved quality among the internal medicine and other specialists.

A position paper published by the Israeli Medical Association (IMA) in 2018 pointed to several problems, including problems in correctly measuring essential indicators (i.e., not all essential indicators can be measured accurately), methodological difficulties in standardizing patients’ health and socioeconomic status, creating incentives for patient selection, creating an incentive to provide unnecessary treatments aimed at influencing indicators’ performance rates, allocation of resources and management effort to actions that are measured at the expense of those that are not are measured. In this position paper the IMA recommended to maintain only 9 quality indicators [19]. However, it should be noted that this is a position paper and not supported by empirical data.

Due to the differences among healthcare systems around the world and the type of quality indicators employed in each country [20, 21], it is difficult to compare findings across countries. There have been calls to align quality indicators across organizations and countries [22, 23]. Furthermore, there is a lack of studies on PCPs’ perceptions on measuring quality indicators in health, specifically in primary care.

Whereas in Israel all indicators are retrieved centrally from the computerized electronic medical record, in other countries, doctors report their performance in quality indicators in writing. In a study conducted in the Netherlands among medical specialists, residents and nurses working in intensive care in 8 hospitals, 66% perceived documenting quality indicator data as unnecessary and 18% perceived them as unreasonable. Unnecessary documentation was perceived as reducing the sense of autonomy. Nevertheless, documentation burden had no effect on the perceived joy in work [24]. Documentation requirements for electronic health records, including quality metrics, compliance and billing have also been reported to contribute to stress and burnout among PCPs [25, 26].

As mentioned above, our findings showed that female PCPs (versus male PCPs), non-Jewish PCPs (versus Jewish ones), and those who are not board certified (versus board-certified PCPs) were more likely to support the Program for Quality Indicators in Community Healthcare and its continuation.

Gender differences in clinical decision-making may stem from implicit biases and historical biases in medical education, which can influence how doctors approach quality care​ [27]. This is supported by findings that female doctors often exhibit more empathizing traits, aligning with programs that prioritize patient-centered or person-centered care [27, 28] However, the differences between male and female physicians in relation to overall performance on quality measures have been shown to be minimal in contemporary settings where advanced clinical decision support and feedback systems are in place. In such environments, the adoption of quality indicator programs tends to be similar across genders​ [29].

There is evidence that minority physicians are more supportive of quality improvement programs that directly tackle health disparities, especially those related to preventive screenings and patient safety, where minority patients often experience lower levels of care​ [30]. Minority physicians frequently report that healthcare systems are less responsive to the needs of diverse patient populations, leading to their greater advocacy for quality indicator programs that address these gaps​ [31].

It is possible that the program helps PCPs who are not board certified to follow important guidelines and emphases in treatment, while this expectation may evoke resistance among specialist PCPs due to perceived reduced autonomy. Studies have suggested that the increasing emphasis on quality indicator programs, particularly under value-based care models, has led to concerns among physicians about a loss of clinical decision-making freedom. These programs, often linked to performance metrics and reimbursement systems, are seen by some doctors as reducing their autonomy by imposing standardized care protocols that may not account for individual patient needs [32]. For example, in a study that examined physicians’ perceptions in Canada, the United States and Norway has found significant concerns about the freedom to make clinical decisions. Physicians working in the United States particularly reported higher levels of perceived autonomy compared to their Canadian and Norwegian counterparts. However, many doctors across these countries felt that these programs limited their ability to spend adequate time with patients and exercise clinical freedom, impacting their job satisfaction and perceptions of quality care​ [33]. Moreover, some researchers argue that while quality indicators can improve patient outcomes, the way they are implemented often reduces physicians’ sense of professional autonomy, potentially leading to burnout and dissatisfaction [34].

### Limitations

Although some of the demographic parameters collected in 2020 were different from those of the 2010 survey (age, country of birth, specialty, type of employment and main type of practice), the analysis was adjusted for these parameters so that the natural differences in populations between the survey timepoints would not affect the results. The survey was done among a random sample of PCPs working in each of the four public HMOs and the results were weighted for the size of the HMOs; therefore, its findings represent the perceptions of Israeli PCPs working in Israel’s public health system. However, a selection bias may be present because the HMOs provided us only with contact details of PCPs and not their characteristics; therefore, it was not possible to compare the attributes of respondents to those who declined to respond. It is not possible to estimate the bias of whether respondents who were more supportive of the program or opposed the program were more inclined to answer the survey. Additionally, in the 2010 survey there were twice as many family medicine specialists compared to internal medicine/other specialists (40.6% versus 20.2%), whereas in 2020 their percentage were almost equal (35.5% versus 29.6%). However, analysis of the responses by professional groups showed similar trends regarding their opinions about the program and their support for the program. As some HMOs added additional internal indicators, the burden perceived by PCPs working in each HMO may be different. To the best of our knowledge, the physicians are not aware which indicators are National Program ones, and which are internal.

The two surveys were conducted 10 years apart, and although the questions were identical, there was a difference in the method for completing the questionnaires: In the 2010 survey, most questionnaires were completed by regular mail, fax or phone and in 2020 most questionnaires were completed online. These different means for survey completion may have led to a social desirability bias among respondents, despite the assurance of anonymity. Last, the study could have been subject to a response bias, which could not have been addressed.

## Conclusions and policy implications

The study indicates that despite a slight decrease, support for the National Program for Quality Indicators in Community Healthcare remains relatively high among PCPs, and most PCPs recognize its importance in improving the quality of patient care. Nevertheless, similar to the findings in 2010, the program seems to increase PCP workload and reduce work satisfaction.

Therefore, it should be evaluated whether the program adversely impacts PCPs’ workload and whether increased workload due to the program affects patient care. Measuring PCPs’ attitudes towards the program by routine surveys may foster greater engagement and satisfaction and help us to understand which changes should be undertaken.

Ultimately, there is an opportunity for program leaders and HMOs to engage in a broader dialogue with PCPs to refine the program’s design and implementation. The cooperative relationship between HMOs and PCPs in Israel offers a strong foundation for constructive discussions that could improve the program and align it with the needs of clinics and physicians, while also addressing broader national health objectives.

## Data Availability

The data presented in this study are available on request from the corresponding author. The data are not publicly available due to privacy restrictions.

## Abbreviations

HMO: Health maintenance organization
NS: Not statistically significant
PCP: Primary care physician
OECD: Organisation for economic co-operation and development
